# Babies in occiput posterior position are significantly more likely to require an emergency cesarean birth compared with babies in occiput transverse position in the second stage of labor: A prospective observational study

**DOI:** 10.1111/aogs.13765

**Published:** 2019-12-12

**Authors:** Nicola Tempest, Steven Lane, Dharani Hapangama, UK Audit Ressearch Trainee Collaborative in Obstetrics, Gynecology (UK‐ARCOG), Wattar Bassel, Tamblyn Jennifer, Parry‐Smith William, Prior Matthew, Chandrasekaran Dhivya, Bansali Shailly Sahu, Boregowda Geethanjali, Dave Fulva, Guiver Julie, Gupta Mohita, Iyasere Cecilia, Jones Kate, Karanam Vijaya, Player Jessica, Bhatt Daxina, Boyes Zoe, Carpenter Ciara, Clarke Helen, Corr Trent, Dauleh Sarah, Egan Jemma, George Smitha, Harris Phillipa, Kerr Robbie, Ramachandran Nira, Ramage Jennifer, Tildesley Rachel, Paterson Kirsty, Akhter Ferdousi, McDougall Anna, Ojukwu Obi, Tvarozkova Katarina, Ramshaw Nicola, Creed Michelle, Danby Kate, Fisher Amy, Grainger Rosie, Hardy Dorota, Lovell Rebecca, McCormick Aiste, Pearce Melissa, Stelling Heidi, Thompson Ruth, Williams Simon, Wonnacott Alison, Yorke Jemma, Kamran Atiyah, Fernandes Jandy, Zafrani Lamia, Conacher Angus, Tannous Dina, Tracey Susan, Manning Louisa, Bain Alex, Braschi Jessica, Dehnel Alexandra, Okano Sayaka, Rayner Eleanor, Day Fiona, Abdou Alaa, Channing Sarah, Hayward Samantha, Ingamells Sarah, O’Sullivan Marie, Jones Samantha, Day Tipswalo, Haque Noreen, Jilani Kiran, Jones Angharad, Pilkington Lisa, Roberts Ruth, Stone Cath, Thomas Caryl, Davey Mark, Berry Janet, Mukherjee Srabini, Nightingale Gemma, Perumalla Margaret, Johnston Catherine, Smyth Sarah, Smotra Grisham, Subba Kamana, Ghosh Mausumi, Gulati Nidhi, Merzougui Sarra, Miti Chimwemwe, Mohamed Mohammad, Nicholson Yulia, Raghavan Radhae, Seetharaman Priyadharshini, Smyth Camille, Stevenson Helen, Syeda Nasreen, Tan Alex, Veal Laura, Zahid Rabia, Madari Sheethal, McCormack Claire, Barlow Catriona, Beatty Laura, D’Sa Anusha, Hendry Fiona, Martin Iain, McAlpine‐Scott Victoria, Short Daniel, Dalton John, Cartland Sarah, Doxford‐Hook Elizabeth, Kakara Sudeepthi, Pettinger Tom, Scott Malcolm, Tibbott James

**Affiliations:** ^1^ Liverpool Women’s Hospital NHS Foundation Trust Liverpool UK; ^2^ Department of Women’s and Children’s Health Institute of Translational Medicine University of Liverpool Liverpool UK

**Keywords:** cesarean section, Kielland forceps, manual rotation, occiput posterior, occiput transverse, rotational delivery, rotational ventouse

## Abstract

**Introduction:**

Malposition complicates 2‐13% of births at delivery, leading to increased obstetric interventions (cesarean section and instrumental delivery) and higher rates of adverse fetal and maternal outcomes. Limited data are available regarding the likely rates of obstetric intervention and subsequent neonatal and maternal outcomes of births with babies in persistent occiput posterior position vs those in persistent occiput transverse position. The UK Audit and Research trainee Collaborative in Obstetrics and Gynecology (UK‐ARCOG) network set out to collect data prospectively at delivery on final mode of delivery and immediate outcomes.

**Material and methods:**

The UK‐ARCOG network collected data on all births with malposition of the fetal head complicating the second stage of labor (n = 838) (occiput posterior/occiput transverse) requiring rotational vaginal operative birth or emergency cesarean to expedite delivery across 66 participating UK National Health Service maternity units over a 1‐month period. The outcomes considered were the need for emergency cesarean section without a trial of instrumental delivery, success of the first method of delivery employed in achieving a vaginal delivery and neonatal/maternal outcomes.

**Results:**

Obstetricians regarded assistance with an operative vaginal delivery method to be unsafe in 15% of babies in occiput posterior position and 6.1% of babies in occiput transverse position, and they were delivered by primary emergency cesarean section. When vaginal delivery was deemed safe (defined as attempted assisted vaginal rotational delivery), the first instrument attempted was successful in 74.4% of occiput posterior babies and 79.3% of occiput transverse babies.

**Conclusions:**

Our data facilitates decision making by obstetricians to increase safety of assisted rotational operative delivery of a malpositioned baby at initial assessment and in counseling women. Until data from a well‐designed randomized controlled trial of instrumental delivery vs emergency cesarean section are available, this manuscript provides contemporaneous national data from a high resource setting within a structured training program, to assist the selection of an appropriate instrument/method for the delivery of a malpositioned baby.

**Abbreviations**
CI: confidence interval
EMCS: emergency cesarean section
KF: Kielland forceps
MROT: manual rotation followed by the use of traction forceps or ventouse
OP: occiput posterior
OR: odds ratio
OT: occiput transverse
pEMCS: second stage cesarean section without a trial of an instrument
RCT: randomized controlled trial
RV: rotational ventouse
UK‐ARCOG: UK Audit and Research trainee Collaborative in Obstetrics and Gynecology

**Key message**

Rotational vaginal operative deliveries are used widely across the UK with good safety data. With no randomized controlled trial available to inform best practice, our data will assist the selection of the appropriate first instrument and method of delivery of a malpositioned baby.

## 1 INTRODUCTION

Fetal malposition is defined as a fetus in a longitudinal lie with the head in occiput posterior (OP) or occiput transverse (OT) position entering the pelvis first.^1^ OT and OP positions account for approximately 20% of all cephalic fetuses at the onset of labor and around 2‐13% at delivery, with a greater proportion occurring in nulliparous women.^2^, ^3^ Persistent malposition has been associated with a longer second stage of labor, postpartum hemorrhage, third and fourth degree perineal lacerations and increased neonatal morbidity, compared with correcting the malposition and delivery in occiput anterior position.^3^, ^4^, ^5^ Although some women will achieve a spontaneous vaginal delivery of a malpositioned fetus without assistance, most require obstetric intervention; thus, malposition increases cesarean section (2‐ to 6‐fold increased risk) and instrumental delivery (1.5‐ to 4‐fold increased risk).^3^

Rotational delivery is defined as an assisted vaginal delivery that initially requires >45º rotation of the fetal head to achieve occiput anterior position, prior to the completion of delivery.^6^ The available methods to assist delivery of the malpositioned baby include the traditional instrumental vaginal rotational delivery methods, rotational ventouse (RV) or Kielland forceps (KF), and the increasingly popular manual rotation, followed by the use of traction forceps or ventouse (MROT)^7^ and second stage cesarean section without a trial of an instrument (pEMCS). The majority of obstetricians consider rotation of the fetal head to be an acceptable obstetric intervention (97%),^8^ but these are high‐risk procedures that require advanced skills, without evidence from randomized trials to guide the decision to attempt either an instrumental delivery or to proceed directly to a pEMCS.^9^

Determining the likely success rate at the initial assessment of a malpositioned fetus will aid obstetricians in deciding the method to employ, counseling patients, the place of delivery (eg, operating theater or delivery room) and the personnel required for the delivery. We therefore aimed to compare the differences in the need for cesarean section, success rates in achieving vaginal deliveries, and other relevant maternal and neonatal outcomes between two cohorts of women: those who required obstetric intervention because of persistent OP and those with persistent OT in obstetrics units across the UK.

## 2 MATERIAL AND METHODS

The UK Audit and Research Collaborative in Obstetrics and Gynecology network (UK‐ARCOG network, a non‐profit‐making, trainee‐led collaborative for research in obstetrics and gynecology in the UK, see Group Authorship) carried out a national prospective service evaluation study. A pilot study was initially undertaken over 1 week in February 2016 in four NHS obstetric units (in three deaneries) endorsing the feasibility of the study. The main national multicenter service evaluation study was conducted according to prospectively registered protocols at each local Research and Development department in NHS obstetrics units over one calendar month (1‐31 May 2016).

**Figure 1:**
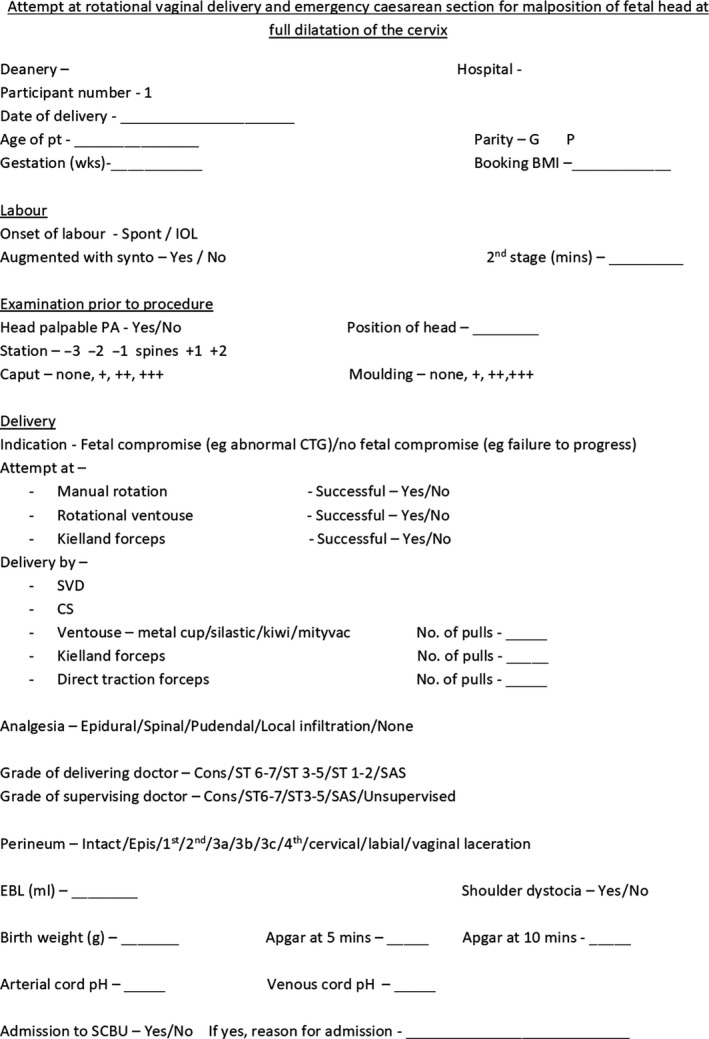
Standardized paper data collection tool. CS, cesarean section; EBL, estimated blood loss; IOL, induction of labor; SCBU, Special Care Baby Unit; SVD, spontaneous vaginal delivery

The UK‐ARCOG recruited 197 specialty trainees (ST1‐7) in obstetrics and gynecology to collect data across 66 NHS hospitals in 12 deaneries across the UK. Each Regional Lead recruited trainees from their local obstetric units, Regional Unit Representatives, who coordinated the collection and completion of a standardized paper data collection tool (Figure 1) during all eligible deliveries by obstetricians performing the deliveries. The Regional Unit Representatives relayed the completed anonymous (containing no patient identifiers) proformas to the Regional Leads, who uploaded the data on to excel files and submitted them for the final analysis. The data from the different regions were merged centrally and the final dataset was entered into an excel database and migrated into statistical package for the social sciences version 21 (IBM Corp, Armonk, NY, USA) for analysis. The research question and outcome measures were developed following discussion, a literature search and a pilot study highlighting the lack of available knowledge to guide clinicians when faced with a malposition in the second stage of labor.

**Figure 2:**
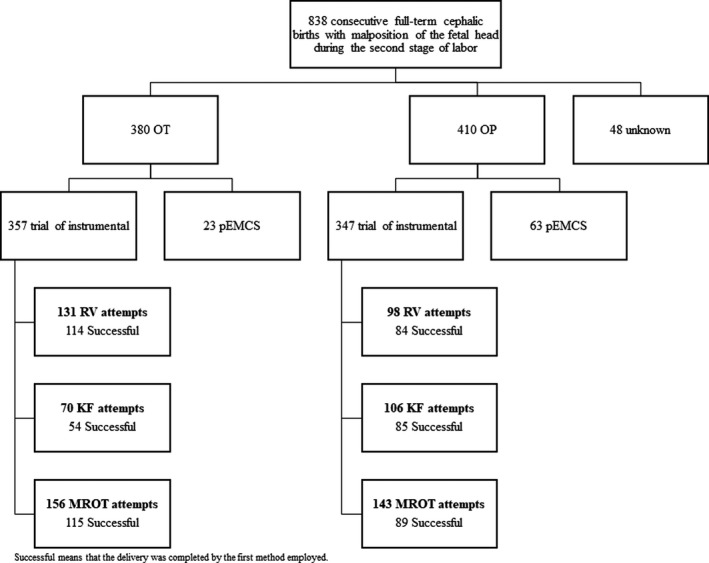
Flowchart of included deliveries. KF, Kielland forceps; MROT, manual rotation followed by the use of traction forceps or ventouse; OP, occiput posterior; OT, occiput transverse; pEMCS, second stage cesarean section without a trial of an instrument; RV, rotational ventouse

All births with malposition of the fetal head complicating the second stage of labor (OP or OT) requiring rotational vaginal operative birth or pEMCS to expedite delivery, were included (Figure 2). Success of the first instrument for MROT was defined as births where MROT was employed to correct malposition in the second stage of labor, followed by the immediate use of either direct traction forceps or non‐rotational ventouse to complete a vaginal birth. If RV or KF was employed after an attempted MROT, that delivery was categorized as an unsuccessful first instrumentation for MROT.

Demographics were collected and the categories of delivery method were RV, KF, MROT and pEMCS. We considered the factors that might influence both the choice of method employed to assist birth and the potential outcomes, eg, a diagnosis of potential fetal compromise vs failure to progress^10^ as an indication for birth will affect neonatal outcomes and may affect the choice of mode of delivery.

We compared the differences in the need for cesarean section, success rates in achieving vaginal deliveries, and other relevant maternal and neonatal clinical outcomes between two cohorts: those who required obstetric intervention because of persistent OP and those with persistent OT.

The demographic and clinical data, stratified by position (OP vs OT), was summarized using means and medians for continuous data and counts and percentages for categorical data. Statistical analysis was undertaken to determine whether position (OP vs OT) was associated with the outcomes using the Chi‐square test, independent sample *t* test and Mann‐Whitney *U* test depending on the distribution of the data. All tests were undertaken at the 5% significance level. Outcomes stratified by both position and method are summarized using mean, medians, standard deviation and interquartile range for continuous variables, depending on their distribution and counts and percentages for categorical data.

### 2.1 Ethical approval

This study, collecting data on current national practice, was exempt from ethical approval according to the NHS Research Ethics Committee.

## 3 RESULTS

A total of 13 890 babies were born at the participating hospitals during the study period. Retrospectively collected mode of delivery data showed 2432 (17.5%) were delivered by planned elective cesarean section, 3195 (23%) births were by EMCS, and 2363 (17%) were operative vaginal births.

Prospective data from 838 consecutive full‐term cephalic births with malposition of the fetal head identified during the second stage of labor were analyzed (for demographics see Table 1). This includes 410 OP and 380 OT (48 unknown) positioned babies: 347 OP babies who had a trial of instrumental and 63 (15.4%) delivered by a pEMCS, compared with 357 OT babies who had a trial of instrumental delivery and 23 (6.1%) who had a pEMCS. Among the 704 deliveries where a vaginal rotational delivery method was attempted, RV (n = 229, 32.5%), KF (n = 176, 25%) and MROT (n = 299, 42.5%) were the first methods used.

**Table 1:** Demographics (for missing data please see Table S1)

	Occiput posterior n = 410	Occiput transverse n = 380	*P* value
Age, mean (SD)	28.5 (5.1)	29.5 (5.3)	0.006^a^
Nulliparous	308 (75.1)	291 (76.6)	0.63^b^
Parous, n (%)	102 (24.9)	89 (23.4)	
Body mass index, mean (SD)	26.5 (5.4)	25.7 (5.3)	0.03^a^
Induction of labor	165 (41.2)	183 (49.3)	0.02^b^
Spontaneous, n (%)	235 (58.8)	188 (50.7)	
Augmented, n (%)			
Yes	181 (44.9)	132 (35.3)	0.006^b^
No	222 (55.1)	242 (64.7)	
2nd stage minutes mean (st dev)	145.8 (67.7)	151.4 (83)	0.37^a^
Head palpable per abdomen n (%)			
No	294 (80.1)	290 (86.8)	0.02^b^
Yes	73 (19.9)	44 (13.2)	
Station, n (%)			
–2/–1	55 (13.5)	19 (5.1)	0.001^b^
Spines/+3	351 (86.5)	356 (94.9)	
Caput, n (%)			
0‐1	260 (63.4)	258 (67.9)	0.35^b^
2‐3	150 (36.6)	122 (32.1)	
Moulding, n (%)			
0‐1	333 (82.6)	320 (85.3)	0.14^b^
2‐3	70 (17.4)	55 (14.7)	
Failure to progress, n (%)	211 (53.8)	210 (55.2)	0.70^b^
Fetal distress, n (%)	181 (46.2)	163 (44.3)	
Analgesia, n (%)			
Spinal	178 (43.7)	137 (36.2)	NA
Epidural	168 (41.3)	176 (46.6)	
Local	27 (6.6)	39 (10.3)	
Pudendal	16 (3.9)	20 (5.3)	
General anesthetic	15 (3.7)	3 (0.8)	
None	3 (0.7)	3 (0.8)	
Birthweight, mean (SD)	3482.6 (525.4)	3521.2 (506.1)	0.29^a^

N/A, there are too many categories to have statistical meaning.

a Independent sample *t *test.

b Chi‐squared test.

### 3.1 Outcomes

OP position was associated with a statistically higher rate of pEMCS and a lower success rate of the first instrument used for vaginal delivery, compared with OT position (15 vs 6.1%, *P* < 0.001). When vaginal delivery was deemed safe, the first instrument attempted was successful in 74.4% of the babies in OP position and 79.3% of the OT babies (*P* = 0.12) (Table 2). After adjusting for body mass index (BMI) and indication for delivery, there was no difference in success rates with OP position compared with OT position (1.38 odds ratio [OR], 95% confidence interval [CI] 0.94‐ 2.01).

**Table 2:** Procedures for delivery according to the malposition and outcomes (for missing data please see Table S1)

	Occiput posterior n = 410	Occiput transverse n = 380	*P* value
pEMCS n (%)	63 (15)	23 (6.1)	<0.001^a^
Attempted assisted vaginal delivery, n (%)	347 (85)	357 (94.9)	
Vaginal delivery attempted, n (%):	258 (74.4)	283 (79.3)	
first instrument success	16 (4.6)	14 (3.9)	
further instrumentation success	60 (17.2)	46 (12.8)	0.12^a^
failed first instrument EMCS further instrument failure and EMCS	13 (3.7)	14 (3.9)	NA
Sphincter injury when vaginal delivery attempted, n (%)			
No	324 (94.5)	340 (95)	0.76^a^
Yes	14 (5.5)	18 (5)	
Estimated blood loss, n (%)			
<1500	392 (96.3)	364 (96.0)	0.84^a^
≥1500	15 (3.7)	15 (4.0)	
Shoulder dystocia when vaginal delivery attempted, n (%)			
No	337 (97.1)	332 (93.8)	0.04^a^
Yes	10 (2.9)	22 (6.2)	
Apgar at 5 min, n (%)			
<7	28 (6.9)	24 (6.3)	0.77^a^
≥7	380 (93.1)	356 (93.7)	
Arterial pH, median (IQR)	7.20 (11)	7.20 (12)	0.53^b^
SCBU, n (%)			
No	376 (92.6)	346 (91.8)	0.66^a^
Yes	30 (7.4)	31 (8.2)	

N/A, There were too many categories to have statistical meaning.

Abbreviations: EMCS, emergency cesarean section; IQR, interquartile range; pEMCS, second stage cesarean section without a trial of an instrument; SCBU, Special Care Baby Unit.

a Chi‐squared test.

b Mann‐Whitney test.

### 3.2 Occiput posterior position

The dataset contained 410 babies in OP position, detailing the methods employed for their delivery and the subsequent outcomes (first instrument success, further instrumentation success, further instrument failure and EMCS, and failed first instrument EMCS) detailed in Table 3. When RV was attempted in the OP position, there was an 85.7% (n = 84) success rate of achieving a vaginal delivery with the first instrument used, and 12.2% (n = 12) failing and having an EMCS. Among attempts to deliver a baby in the OP position, KF resulted in an 80.2% (n = 85) success rate in achieving a vaginal delivery with the first instrument used, 16% (17) of the attempts failing and having an EMCS. When MROT was attempted, there was a 62.4% (n = 89) success rate with first instrument used and 21.7% (31) were unsuccessful, leading immediately to an EMCS.

**Table 3:** Outcomes by methods with position

	Occiput posterior n = 410				Occiput transverse n = 380			
	RV n = 98	KF n = 106	MROT n = 143	pEMCS n = 63	RV n = 131	KF n = 70	MROT n = 156	pEMCS n = 23
First instrument success, n (%)	84 (85.7)	85 (80.2)	89 (62.4)	63 (100)	114 (87.0)	54 (77.1)	115 (73.7)	23 (100)
Further instrumentation success, n (%)	1 (1.0)	4 (3.8)	11 (7.7)		3 (2.3)		11 (7.1)	
Further instrument failure and EMCS, n (%)	1 (1.0)		12 (8.4)		1 (0.8)	1 (1.4)	12 (7.7)	
Failed first instrument EMCS, n (%)	12 (12.2)	17 (16.0)	31 (21.7)		13 (9.9)	15 (21.4)	18 (11.5)	
Sphincter injury, n (%)								
No	89 (91.8)	99 (94.3)	136 (96.5)	61 (100.0)	128 (97.7)	64 (91.4)	147 (94.2)	23 (100.0)
Yes	8 (8.2)	6 (5.7)	5 (3.5)		3 (2.3)	6 (8.6)	9 (5.8)	
EBL, n (%)								
<1500	97 (99.0)	101 (96.2)	139 (97.2)	53 (89.8)	125 (95.4)	67 (97.1)	144 (92.9)	22 (95.7)
≥1500	1 (1.0)	4 (3.8)	4 (2.8)	6 (10.2)	6 (4.6)	2 (2.9)	11 (7.1)	1 (4.3)
Shoulder dystocia, n (%)								
No	95 (96.9)	101 (95.3)	141 (98.6)	63 (100)	122 (95.3)	65 (92.9)	144 (92.9)	23 (100.0)
Yes	3 (3.1)	5 (4.7)	2 (1.4)		6 (4.7)	5 (7.1)	11 (7.1)	
Birthweight (g), mean (SD)	3475.2 (488.5)	3540.4 (475.8)	3389.8 (541.8)	3611.1 (602.5)	3432.6 (487.4)	3529.1 (542.1)	3561.3 (501)	3731.7 (460.9)
Apgar at 5 min, n (%)								
<7	4 (4.1)	7 (6.6)	9 (6.3)	8 (13.1)	10 (7.6)	2 (2.9)	10 (6.4)	2 (8.7)
≥7	94 (95.9)	99 (93.4)	134 (93.7)	53 (86.9)	121 (92.4)	68 (97.1)	146 (93.9)	21 (91.3)
Arterial pH, mean (SD)	7.20 (0.10)	7.19 (0.08)	7.20 (0.08)	7.22 (0.10)	7.20 (0.08)	7.17 (0.11)	7.19 (0.10)	7.19 (0.08)
SCBU, n (%)								
No	91 (92.9)	132 (93.0)	132 (93.0)	54 (90.0)	116 (90.6)	64 (91.4)	145 (92.8)	21 (91.3)
Yes	7 (6.7)	10 (7.0)	10 (7.0)	6 (10.0)	12 (9.4)	6 (8.6)	11 (7.1)	2 (8.7)

Abbreviations: EBL, estimated blood loss; EMCS, emergency cesarean section; KF, Kielland forceps; MROT, manual rotation followed by the use of traction forceps or ventouse; pEMCS, second stage cesarean section without a trial of an instrument; RV, rotational ventouse.

### 3.3 Occiput transverse position

A total of 380 babies in the OT position were included in the dataset with a detailed description of their delivery method and relevant outcomes illustrated in Table 3. RV attempted in the OT position was associated with an 87% (n = 114) success rate with the first instrument used, with 9.9% (13) failing and having an EMCS. When attempts were made to deliver babies in OT position with KF, the success rate with first instrument was 77.1% (n = 54), with 21.4% (n = 15) failing and having an EMCS; attempts at MROT deliveries were 73.7% (n = 115) successful with the first instrument, and 11.5% (18) attempts failing and having an EMCS.

MROT was the most commonly employed rotational vaginal delivery method for both OP and OT, with 42.5% (n = 299/704) attempted deliveries using this method. MROT was also associated with the lowest success rates for the first instrument, achieving a vaginal delivery (OP 62.4% and OT 73.7%, Table 3). Additionally, the subsequent successful use of a second rotational delivery instrument was relatively high with MROT in both OP (7.7%) and OT (7.1%) deliveries. Overall, RV was associated with the highest rates of success regardless of the fetal position.

When pEMCS was compared directly with all attempts at vaginal rotational deliveries, it was found to have a higher incidence of massive obstetric hemorrhage (8.5 vs 3.3%, *P* = 0.02). Although there was an increased incidence of low Apgar <7 at 5 minutes (11.9 vs 6%, *P* = 0.04), this was not associated with a significant increase in Special Care Baby Unit admission (9.5 vs 7.6%, *P* = 0.52) (see Table S2).

## 4 DISCUSSION

This large, consecutive national series of rotational operative deliveries in the second stage of labor from the UK, found that OP position was associated with a statistically higher requirement for pEMCS when compared with OT position. The success of the first instrument when vaginal delivery was attempted (74 vs 79%, *P* = 0.12) was not statistically significant. We observed trends suggesting that MROT is the most prevalent method employed by obstetricians in NHS obstetrics units in the UK to assist in a malposition delivery for both OP and OT positions. Interestingly, this method was also associated with the lowest success rates and presented no additional benefit with regard to maternal or neonatal morbidity. OP position was associated with significantly higher stations of the head, which were more likely to be palpable per abdomen, than was the OT position. This could help explain the slightly higher chance of failure of both RV and MROT in OP position compared with those deliveries attempted in OT position. Conversely, KF used in OP position was more likely to be successful than OT position, suggesting potential inherent differences in these methods when used for OP or OT deliveries. For example, KF may be challenging to use on an OT baby, particularly when the head is low, which may encourage the obstetricians to tend towards alternative options.

The strengths of our study include being the largest reported, prospectively collected series of consecutive deliveries complicated by malposition in the second stage of labor, illustrating obstetric practice within a high‐resource setting where assistance is provided by highly skilled obstetricians. We included all methods suitable for the delivery of malpositioned babies at full dilation which were omitted in previous studies, for example, MROT and pEMCS.^11^ Furthermore, the prospective data, collected directly by the operating obstetrician immediately after delivery, prevented recall bias associated with previously published, retrospectively collected data. At this time, with no randomized controlled trials (RCT) to guide clinicians when confronted with malposition in the second stage of labor, the data presented here are an invaluable addition to the current literature.

A limitation of this study was that it was not powered to examine rare outcomes such as neonatal death and only allowed the assessment of short term/immediate outcomes and complications, since no long‐term follow‐up data were collected. However, our data provide information to calculate sample size for future studies to detect significant differences in neonatal and maternal complications. For example, if an RCT were to be completed comparing pEMCS vs rotational instrumental delivery, to detect differences in neonatal complications, such as Special Care Baby Unit admission, it would require over 15 000 births per group to detect a 1% reduction in Special Care Baby Unit admission at the 5% significance level and with 90% power, whereas anal sphincter damage differences can be examined with a study including 10 000 births per group, and again would detect a 1% reduction in sphincter injury at the 5% significance and 90% power. Such larger studies would naturally require a multicenter, international RCT in the future. A further limitation of our study was the retrospective collection of the total numbers of all deliveries in the participating units, during the time period; thus this particular aspect should be interpreted with caution.

Recently published retrospective data from British Columbia, Canada, have suggested that attempted mid‐cavity operative vaginal delivery is associated with higher rates of severe perinatal morbidity/mortality compared with pEMCS.^12^ Although that series did include some malpositioned babies, the authors did not depict the outcomes related to this important high‐risk sub‐category. The rates reported by the Canadian group are clearly different to our data, which agree with many previous reports from the UK.^5^, ^11^, ^13^, ^14^ For example, the high rates of anal sphincter damage reported with the Canadian series, ranging from 23% with forceps to 10.3% with ventouse deliveries, are not comparable to the rates reported from UK or from New Zealand.^6^, ^7^, ^15^ The increased incidence of maternal hemorrhage associated with pEMCS when compared with the vaginal operative delivery methods is well established 5, 7, 11, 16, 17 but Muraca et al report that second stage cesarean section has a much lower rate of maternal hemorrhage than instrumental delivery (cesarean section 4.62% vs ventouse 13.9% and forceps 21.2%). This is in stark contrast to our series, which showed pEMCS was associated with a statistically higher incidence of massive obstetric hemorrhage (*P* = 0.02) and Apgar <7 at 5 minutes (*P* = 0.04), and increased Special Care Baby Unit admissions when compared with all attempted rotational vaginal deliveries. Our data therefore suggest that although some rotational (whether instrumental or manual) vaginal deliveries fail, it is still a safe and valid option in many selected cases.

Previous studies have indicated KF/RV to be associated with a higher incidence of adverse outcomes when compared with direct traction instruments; therefore the higher rates reported by the Canadian group, which contain a mixture of both rotational and direct traction instruments, are likely to reflect the specific practice in Canada and cannot be generalized to operative delivery practice worldwide. Interestingly, Muraca et al also suggested that deliveries with presumed lack of dystocia (fetal distress being the indication) were associated with a lower incidence of severe maternal and fetal outcomes when compared with pEMCS.^12^ This further highlights that correct patient selection is imperative for the best outcome with the selected method of delivery. In this national series from the UK, obstetricians appear to choose a particular method of delivery for a specific patient population. This is exemplified by the more successful use of KF in babies in OP position, which was more commonly associated with caput and moulding; whereas OT positioned babies who were more likely to be at a lower station (Table 2) were successfully delivered with RV with a much lower rate of adverse outcomes.

Overall, with our UK‐wide national dataset, we propose that in a high‐resource setting within a structured training program, where a high volume of operative vaginal delivery is practiced in a standardized manner, obstetricians can acquire and retain skills to perform rotational vaginal operative deliveries to achieve safer outcomes.

### 4.1 Implications for research

A well‐designed RCT of instrumental delivery compared with pEMCS when malposition is diagnosed in the second stage of labor and instrumental delivery is deemed, by an experienced obstetrician, to be safe to proceed to, will allow confirmation of these trends in the future (the Health Technology Assessment [HTA] have called for this data to be produced).

### 4.2 Implications for practice

It is reasonable to offer rotational vaginal delivery in selected cases and this dataset, which reflects current clinical practice, can be very helpful in providing women with risk and success rate information to aid informed choice.

## 5 CONCLUSION

The knowledge that when compared with OT babies, OP position is associated with a statistically higher requirement for pEMCS and an apparently lower success of the baby being delivered by the first instrument attempted, will help with risk stratification, patient counseling and delegation of expertise, eg, the availability of senior input. Until data are available from a well‐designed RCT, we hope this manuscript will assist in the selection of an appropriate instrument/method for the delivery of a malpositioned baby and allow a senior obstetrician to make an educated decision regarding the necessity for their presence for a particular delivery. It will also provide clinicians with statistics reflecting current practice to inform counseling and provide information.

## CONFLICT OF INTEREST

The authors declare that they have no conflict of interest.

## APPENDIX 1

UK‐ARCOG core committee: Bassel Wattar, Jennifer Tamblyn, William Parry‐Smith, Matthew Prior, Dhivya Chandrasekaran.

East Midlands Regional Unit Representatives (RUR): Shailly Sahu Bansali, Geethanjali Boregowda, Fulva Dave, Julie Guiver, Mohita Gupta, Cecilia Iyasere, Kate Jones, Vijaya Karanam, Jessica Player.

Merseyside (RUR): Daxina Bhatt, Zoe Boyes, Ciara Carpenter, Helen Clarke, Trent Corr, Sarah Dauleh, Jemma Egan, Smitha George, Phillipa Harris, Robbie Kerr, Nira Ramachandran, Jennifer Ramage, Rachel Tildesley.

North East Thames Regional Lead: (RL) Kirsty Paterson, (RUR) Ferdousi Akhter, Anna McDougall, Obi Ojukwu, Katarina Tvarozkova.

Northern England: (RL) Nicola Ramshaw, (RUR) Michelle Creed, Kate Danby, Amy Fisher, Rosie Grainger, Dorota Hardy, Rebecca Lovell, Aiste McCormick, Melissa Pearce, Heidi Stelling, Ruth Thompson, Simon Williams, Alison Wonnacott, Jemma Yorke.

North Scotland: (RL) Atiyah Kamran, (RUR) Jandy Fernandes.

North West: (RL) Lamia Zafrani, (RUR) Angus Conacher, Dina Tannous, Susan Tracey.

Peninsula: (RL) Louisa Manning, (RUR) Alex Bain, Jessica Braschi, Alexandra Dehnel, Sayaka Okano, Eleanor Rayner.

Severn: (RL) Fiona Day, (RUR) Alaa Abdou, Sarah Channing, Samantha Hayward, Sarah Ingamells, Marie O’Sullivan.

Wales: (RL) Samantha Jones, (RUR) Tipswalo Day, Noreen Haque, Kiran Jilani, Angharad Jones, Lisa Pilkington, Ruth Roberts, Cath Stone, Caryl Thomas.

Wessex: (RL) Mark Davey, (RUR) Janet Berry, Srabini Mukherjee, Gemma Nightingale, Margaret Perumalla, Catherine Johnston, Sarah Smyth.

West Midlands: (RL) Grisham Smotra, Kamana Subba, (RUR) Mausumi Ghosh, Nidhi Gulati, Sarra Merzougui, Chimwemwe Miti, Mohammad Mohamed, Yulia Nicholson, Radhae Raghavan, Priyadharshini Seetharaman, Camille Smyth, Helen Stevenson, Nasreen Syeda, Alex Tan, Laura Veal, Rabia Zahid.

West Scotland: (RL) Sheethal Madari, Claire McCormack, (RUR) Catriona Barlow, Laura Beatty, Anusha D’Sa, Fiona Hendry, Iain Martin, Victoria McAlpine‐Scott, Daniel Short.

West Yorkshire: (RL) John Dalton, (RUR) Sarah Cartland, Elizabeth Doxford‐Hook, Sudeepthi Kakara, Tom Pettinger, Malcolm Scott, James Tibbott.
