# Supplementary material for: Babies in occiput posterior position are significantly more likely to require an emergency cesarean birth compared with babies in occiput transverse position in the second stage of labor: A prospective observational study
Source: Acta Obstet Gynecol Scand. 2019 Dec 12;99(4):537–45. doi: 10.1111/aogs.13765 (PMC7154761; doi:10.1111/aogs.13765)
Supplement: Supplementary file 1 [file AOGS-99-537-s001.docx]

Table S1. Information regarding missing data.

| ***Variable*** | ***Occiput posterior*** | ***Occiput transverse*** |
| --- | --- | --- |
| Age | 4 | 5 |
| BMI | 13 | 11 |
| Induction of labor | 10 | 9 |
| Augmented | 7 | 6 |
| 2^nd^ stage minutes | 100 | 12 |
| Head palpable per abdomen | 43 | 46 |
| Station | 4 | 5 |
| Moulding | 7 | 5 |
| Failure to progress /fetal distress | 18 | 16 |
| Analgesia | 3 | 2 |
| Birthweight | 4 | 7 |
| Sphincter injury | 9 | 0 |
| Estimated blood loss | 3 | 1 |
| Shoulder dystocia | 0 | 4 |
| Apgar at 5 | 2 | 0 |
| Arterial pH | 69 | 78 |
| Special Care Baby Unit | 4 | 3 |
