# Supplementary material for: Babies in occiput posterior position are significantly more likely to require an emergency cesarean birth compared with babies in occiput transverse position in the second stage of labor: A prospective observational study
Source: Acta Obstet Gynecol Scand. 2019 Dec 12;99(4):537–45. doi: 10.1111/aogs.13765 (PMC7154761; doi:10.1111/aogs.13765)
Supplement: Supplementary file 2 [file AOGS-99-537-s002.docx]

Table S2. All instrumental attempts versus second stage cesarean section without a trial of an instrument (pEMCS).

|  | Instrumental | pEMCS | Significance |
| --- | --- | --- | --- |
| Hemorrhage n (%) <=1500  >1500 | 629 (96.7)  23 (3.3) | 75 (91.5)  7 (8.5) | P=0.02^1^ |
| Apgar at 5 mins n (%) < 7  >= 7 | 42 (6.0)  662 (94.0) | 10 (11.9)  74 (88.1) | P=0.04^1^ |
| SCBU admission n (%) No  Yes | 645 (92.4)  53 (7.6) | 75 (90.5)  8 (9.5) | P=0.52^1^ |

^1^Chi-squared test

SCBU; Special Care Baby Unit
